# Numerical study on all-optical modulation characteristics of quantum cascade lasers

**DOI:** 10.3762/bjnano.13.88

**Published:** 2022-09-23

**Authors:** Biao Wei, Haijun Zhou, Guangxiang Li, Bin Tang

**Affiliations:** 1 Key Laboratory of Optoelectronic Technology & Systems, Ministry of Education, Chongqing University, Chongqing 400044, China

**Keywords:** all-optical modulation, dielectric nanostructures, high refractive index materials, numerical study, quantum cascade lasers

## Abstract

To explain the phenomenon of all-optical modulation of quantum cascade laser (QCL), and explore the physics in QCL’s gain medium which consists of multiple of dielectric nanostructures with high refractive index under light injection, we modified the 1½-period model to calculate values of electron population and lifetime in each subband which is separated by the nanostructures, optical gain, current and number of photons in the cavity of a mid-infrared QCL modulated with near-infrared optical injection. The results were consistent with an experiment, where the injected light increases the electron population and lifetime, but does not affect the optical gain obviously. Our study can be helpful for optimizing its use and dielectric nanostructure design.

## Introduction

The quantum cascade laser (QCL) was invented nearly 30 years ago [1], and its cavity consists of multiple nanostructures, which are grown by molecular beam epitaxy (MBE) [1]. It has been widely used in the fields of free space optical communication [2–3], gas detection [4–5], and biological research [6–7]. Because the QCL is a narrow linewidth and high-power laser working in the mid-infrared to terahertz band, it can cover most of the gas molecular-fingerprint absorption spectrum and atmospheric transmission window, and it will not damage organisms. Modulation of QCLs is an effective method of suppressing low-frequency noise and improving the signal-to-noise ratio. Various approaches to modulation have been reported, including thermal [8], electrical [9], acousto-optic [10], Faraday rotation effect spectroscopy [11], and all-optical methods [12]. The unipolar characteristics of QCLs provide them with unique advantages for all-optical modulation. All-optical modulation can avoid parasitic effects, and the modulation frequency can reach 60 GHz [13]. Moreover, all-optical modulation can directly alter the carrier distribution and is highly efficient. However, the mechanism of all-optical modulation is very complex. If we could effectively describe all-optical modulation of QCLs by numerical simulation, it would be very helpful for optimizing its use and dielectric nanostructure design. Although there has been much research on all-optical modulation of QCLs, only optical injection locking [14] and quenching [15–16] can currently provide one-sided numerical support. Based on the classical 1½-period model of QCLs [17], a numerical calculation method for all-optical modulation of QCLs is proposed here. By considering the change of carrier distribution in the active region of a QCL caused by the injected light, the results of all-optical modulation experiments can be reasonably explained. These findings provide support for further research on all-optical modulation of QCLs.

## Results and Discussion

### Numerical approach

To examine the all-optical modulation of QCLs, we must consider the output characteristics of QCLs. In the four-level system, the carrier distribution is described by the full rate equations (FRE) [18] as Equations 1–4 shows, where *n*_0_ is the cavity index, *g*_c_ is the gain cross section, *c* is the speed of light. Γ_P_ is the optical confinement factor per stage, α is the laser total loss. *N*_P_ the total number of stages, 1/τ_k,i_ is the rate of electron scattered from subband *k* to subband *i*, 1/τ_sp_ is the spontaneous emission rate of the upper laser subband, α is the cavity absorption coefficient, β is the rate of spontaneous emission getting into the laser modes, *n*_k_ is the *k*-th subband population, *S* is the photon population in the cavity, and the laser upper and lower subbands are denoted by Equation 1 and Equation 2, respectively. The numerical methods solving FRE are excellently recorded in [18]. By taking into account the effects of optical injection [19] and electron temperature [20–21], the full rate equation is modified to obtain a more accurate numerical simulation of the output characteristics of QCLs. In all-optical modulation, light directly illuminates the facet of the active region of the QCL. Injection of a large amount of energy will inevitably change the original energy balance and carrier distribution and, of course, generate heat. The injected light will excite the valence band electrons to transition to the conduction band and produce electron-hole pairs. The lifetimes of electrons and holes are quite different [22] and result in accumulation of holes, and then, in order to maintain electrical neutrality of the laser, the electrons in the current are added to the conduction band [23]. Moreover, the fluctuation of threshold current caused by the light injection is around tens of mA [19], which is much smaller than the threshold current. Therefore, all-optical modulation can only be achieved when the QCL works near or above the threshold. It is worth noting that, when the wavelength of the modulating light is close to the band gap of the active region of the QCL, most of the injected light energy excites the electrons in the valence band to transition to the conduction band and then to the upper laser subband, thereby increasing the power of the modulating light. However, with a decrease in the modulated laser wavelength, its energy becomes greater than the band gap between the valence band and the lower laser subband of the conduction band of the QCL active region, with the excess energy exciting the electrons to a higher energy level or high kinetic state (high kinetic state means high electron temperature) and generating heat through electron–electron and electron–lattice scattering, which suppresses the laser output. And such a compression can be explained by the hot electron backfilling effect [20,24–25], which is caused by an increase of the electron temperature. The electrons with high kinetic state increase the number of electrons in the lower laser subband by backfilling and inhibit the population inversion. As a result, the light output is suppressed.


[3] $$\frac{dn_{i}}{dt}=-n_{i}\sum_{k\neq i}\frac{1}{\tau_{i,k}}+\sum_{k\neq i}\frac{n_{k}}{\tau_{k,i}}$$

[4] $$\frac{dn_{4}}{dt}=-n_{4}\sum_{k\neq 4}\frac{1}{\tau_{4,k}}+\sum_{k\neq 4}\frac{n_{k}}{\tau_{k,4}}-S\Gamma_{\text{P}}\frac{c}{n_{0}}g_{\text{c}}(n_{4}-n_{3})$$

[2] $$\frac{dn_{3}}{dt}=-n_{3}\sum_{k\neq 3}\frac{1}{\tau_{3,k}}+\sum_{k\neq 4}\frac{n_{k}}{\tau_{k,3}}+S\Gamma_{\text{P}}\frac{c}{n_{0}}g_{\text{c}}(n_{4}-n_{3})$$

[1] $$\frac{dS}{dt}=S\left[N_{\text{P}}\Gamma_{\text{P}}\frac{c}{n_{0}}g_{\text{c}}(n_{4}-n_{3})-\frac{c}{n_{0}}\alpha \right]+N_{\text{P}}\beta \frac{n_{4}}{\tau_{\text{sp}}}$$

**Figure 1:**
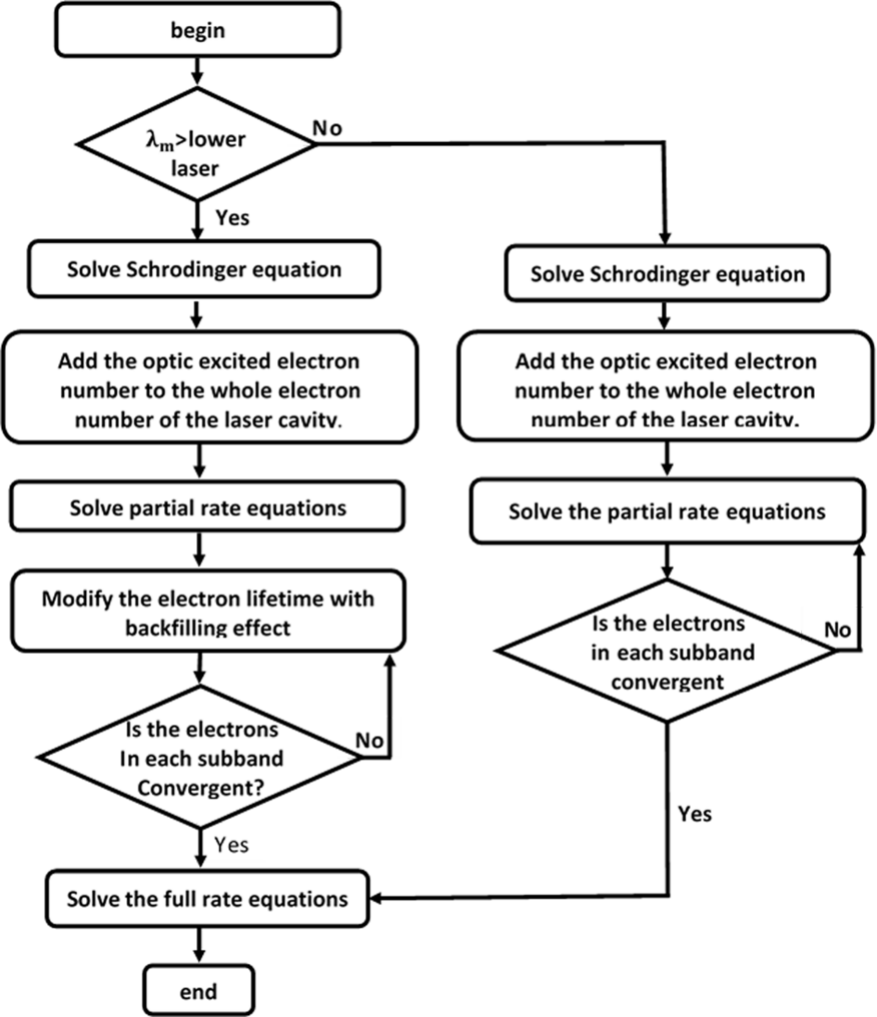
An algorithm for solving the scattering rate and electron distribution for each subband using all-optical modulation.

In this paper, we further modify the rate equation to facilitate a numerical study of the all-optical modulation of QCLs. A flow chart of the numerical calculations for the all-optical modulation of QCLs is shown in Figure 1. Here, we modify our numerical model in two cases. The first case is when modulating the laser wavelength to greater than the lower laser subband of the active region of the QCL. The injected laser will then not be able to excite the electrons in the valence band to the laser subband of the conduction band, and the transition of electrons in the conduction band will be affected by the electric field. Therefore, we can allocate all the photoexcited electrons to the conduction band, and obtain the electron number of each subband of the conduction band by solving the partial rate equations (PRE) [17]. Whether the scattering rate and electron population of each subband can be further calculated by the FRE is judged by whether or not the electrons of each subband converge. Numerical studies of the FRE involved here have been previously reported [18]. The second case is when the modulating laser wavelength is less than that of the lower laser subband of the active region of the QCL. The injected laser light may then excite the electrons to the laser subband or high kinetic state, which requires correct allocation of the photoexcited electrons to each subband; the fluctuation of the electron temperature in the conduct band is an important parameter for characterizing this change. First, the photoexcited electrons are evenly distributed into each subband to complete the initial conditions for the calculations. Through multiple iterations during which the electron number of each subband converges, the electron distribution is approached by energy conservation and the hot electron backfilling effect which increase the lifetime of electrons in the lower laser energy level as described in Equation 5, and finally the scattering rate and number of electrons in each subband are obtained.

The number of photoexcited electrons, which is closely related to the number and area density of photons, forms the basis of the subsequent numerical calculation. When the modulated QCL is illuminated on the facet of its active region at an angle of 30°, almost all the laser energy is absorbed [19], so we disregard this loss, and the initial photoexcited electrons are generated from the valence band.

Furthermore, the number of photons produced during the hole lifetime τ_p_ can be described as:


[6] $$n_{\text{t}}=\frac{P\tau_{\text{p}}\lambda_{\text{inj}}}{ch},$$

where *c* is the speed of light, λ_inj_ is the wavelength of the injected light, *h* is Planck’s constant, and *P* is the power of the modulated laser.

Then, the area density of photons is obtained as:


[7] $$\rho =\frac{n_{\text{t}}}{lw}=\frac{P\tau_{\text{p}}\lambda_{\text{inj}}}{chlw},$$

where *l* is the length and *w* is the width of the laser cavity. All these parameters are known except τ_p_, and τ_p_ can be obtained from the experimental data in reference [19], which gives the relationship between Δλ and injection power; Δλ is the variation in laser wavelength λ caused by optical injection. The relationship between Δλ and the refractive index *n*_0_ can be described as


[8] $$\Delta \lambda =\lambda \frac{\Delta n_{0}}{n_{0}},$$

where Δ*n*_0_ is the variation in *n*_0_.

We know that Δ*n*_0_ is induced by electron-hole pairs, and can be described by the following equation [12]:


[9] $$\Delta n_{0}=-\frac{e^{2}\lambda^{2}}{8\pi^{2}c^{2}\varepsilon_{0}n_{0}}\left(\frac{N}{m_{\text{e}}}+\frac{P}{m_{\text{p}}}\right).$$

Combining Equations 2, 3 and 4, we obtain the following:


[10] $$\Delta n_{0}=-\frac{e^{2}\lambda^{2}}{8\pi^{2}c^{2}\varepsilon_{0}n_{0}}\frac{P\tau_{\text{p}}\lambda_{\text{inj}}}{chlwh}\left(\frac{1}{m_{\text{e}}}+\frac{1}{m_{\text{p}}}\right),$$

which yields:


[11] $$\tau_{\text{p}}=-\frac{\Delta \lambda n_{0}8\pi^{2}c^{2}\varepsilon_{0}n_{0}chlw}{e^{2}\lambda^{2}P\lambda_{\text{inj}}\left(\frac{1}{m_{\text{e}}^{*}}+\frac{1}{m_{\text{p}}^{*}}\right)},$$

where *e* is the charge of an electron, λ is the wavelength of the laser, *N* and *P* are the numbers of the electrons and holes, ε_0_ is the permittivity in a vacuum, and 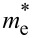 and 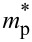 are the effective electron and hole masses, respectively.

When the wavelength of the injected light is 820 nm, the electrons in the cavity are heated and enhance the backfilling effect, which increases the lifetime of electrons in the lower laser energy level (A3) as described by the following equation [20]:


[5] $$\frac{1}{\tau_{3,i}}=\frac{1}{\tau_{3,i}^{'}}-\frac{1}{\tau_{\text{bf}}}\left(e^{-\frac{E_{\text{bf}}}{K_{\text{b}}T}}-e^{\frac{E_{\text{bf}}}{K_{\text{b}}\left(T+\Delta T\right)}}\right)\text{, }i=1,2\;,$$

where *T* is the electron temperature without injected light, Δ*T* is the variation in electron temperature induced by the injected light, *E*_bf_ is the backfilling energy, and τ_bf_ is the backfilling lifetime. τ_3,i_ and τ’_3,i_ are the electron lifetimes in A3 with and without injected light, respectively, and *K*_b_ is the Boltzmann constant. To determine Δ*T*, we assume that all the energy of the optically excited electrons, except those that overcome the bandgap, converts to the kinetic energy of the electrons in the cavity. So the kinetic energy of a single optically excited electron *E* can be described by the following function (it can be verified by the well-known Fermi–Dirac distribution function):


[12] $$E=K_{\text{b}}\Delta T.$$

And *E* can be described as


[13] $$E=\frac{c}{\lambda_{\text{inj}}}h-E_{\text{g}},$$

where *E*_g_ is the bandgap. So the average variation in electron temperature can be described as


[14] $$\Delta T=\frac{En_{\text{inj}}}{K_{\text{b}}\left(n_{\text{inj}}+N_{\text{e}}\right)},$$

where *n*_inj_is the number of optically excited electrons, and *N*_c_ is the number of electrons in the cavity without optical injection.

The values of the key device parameters used in analyzing are summarized in Table 1.

**Table 1:** List of analyzing parameters.

Parameter	Value	Unit	Parameter	Value	Unit

Γ_P_	0.8	–	*E*_bf_	0.1616	eV
*N*_P_	35	–	*N*_c_	2.04 × 10^11^	1/cm^2^
α	23.336	1/cm	*E*_g_	0.8	eV
*n*_0_	3.4	–	τ_p_	8	ns
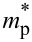	0.4	–	*l*	1.358 × 10^−3^	m
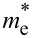	0.042	–	*w*	1.4 × 10^−5^	m
*T*	300	K			

### Simulation

**Figure 2:**
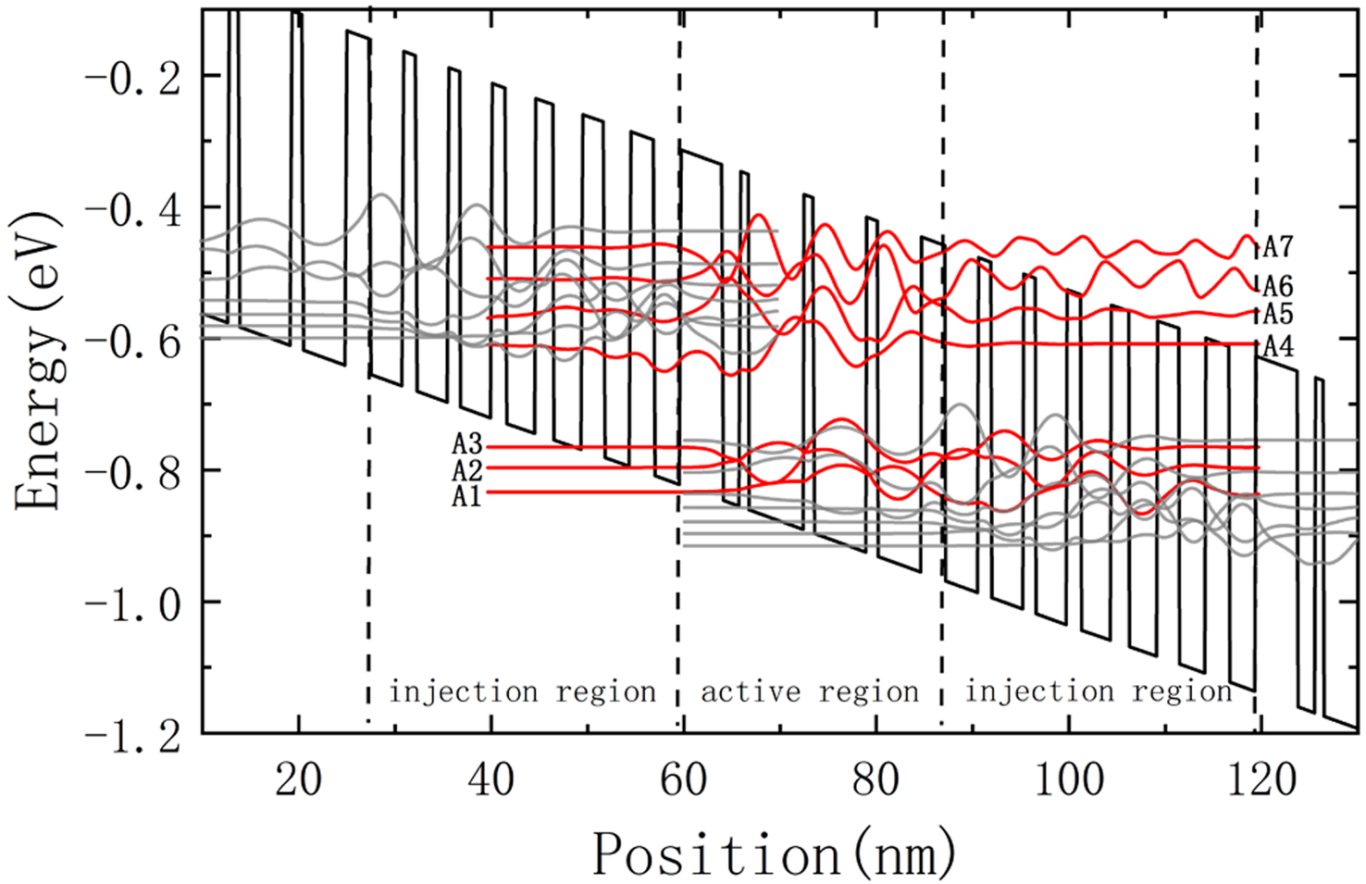
Calculated conduction subbands and moduli squared of relevant wave functions with a 53 kV/cm DC bias.

In this simulation, the laser we studied is the same as in [26], which is a standard 35-stage In_0.52_Al_0.48_As/In_0.53_Ga_0.47_As type-I four-level Fabry–Perot QCL based on a two-phonon-resonance design [27]. Current injection efficiency is defined as the ratio of current to total current injected into the upper subband of the QCL in the active region, which is close to 56%. The QCL total optical loss is assumed to be 23.3 cm^−1^, including a 14.3 cm^−1^ waveguide loss [27] and a 9 cm^−1^ mirror loss for a waveguide refractive index of 3.4. Although there are many bound states in the active region, most electrons remain in several low energy levels. So seven confined subbands are sufficient for the calculation, and here *Ai* (*i* = 1, 2, 3, 4, 5, 6, 7) represents the *i*-th subband in the active region, as shown in Figure 2. Our simulation used an external bias electrical field of 53 kV/cm, which was above the threshold value, and the temperature of the electrons and cavity were 300 K and 30 K, respectively.

### Electron population

**Figure 3:**
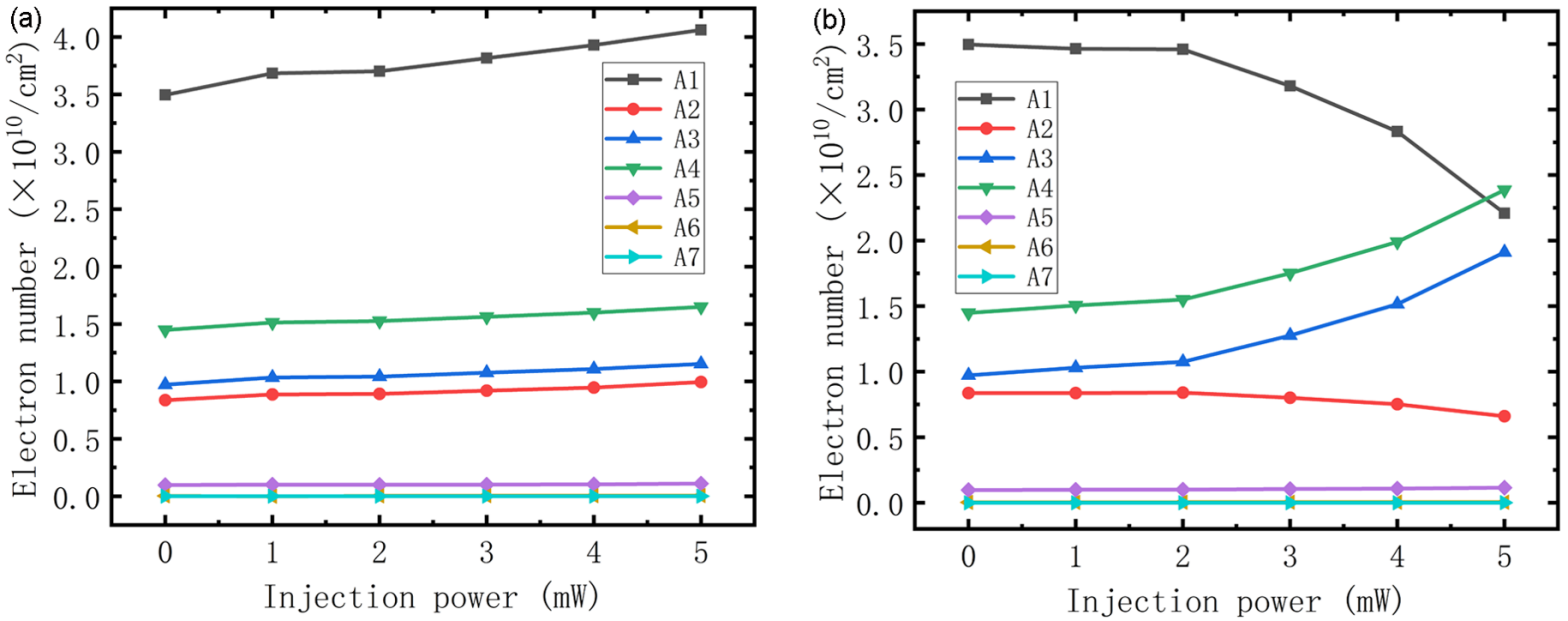
Numbers of electrons in each subband using optical injection at wavelengths of 1550 nm (a) and 820 nm (b).

Figure 3 shows the number of electrons in subbands A1–A7 using an optical injection power of 1–5 mW for wavelengths of 1550 nm (a) and 820 nm (b). In Figure 3a, because the injected light of wavelength 1550 nm increases the number of electrons by accumulating holes, the electron numbers in each subband increase as injection power increases. But electron numbers in subbands A5–A7 are not much different than A1–A4, which results from there being fewer electrons in A5–A7 than A1–A4. In many other studies [17–18,20–21], electrons in A5–A7 have been largely ignored, and similarly, the electron number in A1 is much larger than in the other energy levels, so the variation in the number of electrons in A1 is much larger than in A2–A7. The number of electrons in A2 is lower than in A3 and A4 because it functions as an excessive subband that accelerates the transition of electrons from A3 to A1 [26]. There are some differences in electron numbers of A1–A4 when the wavelength of the injected light is 820 nm, as shown in Figure 3b. First, compared with the behavior in Figure 3a, electron numbers in A3 and A4 increased to a greater extent with increasing injection power. This is a result of a backfilling effect which increased the lifetime of electrons, and hence their number, in A3, resulting in fewer electrons transitioning from A4 to A3 and increasing the number of electrons in A4, leading to the variation in numbers of electrons in A4 with injection power being almost the same as that for A3. Moreover, as the lifetime of electrons in A3 increases, the time that electrons spend in A3 becomes longer, which restrains electrons from transitioning from A3 to A2 and A1, resulting in the numbers of electrons in A1 greatly decreasing with injection power.

### Electron lifetime

**Figure 4:**
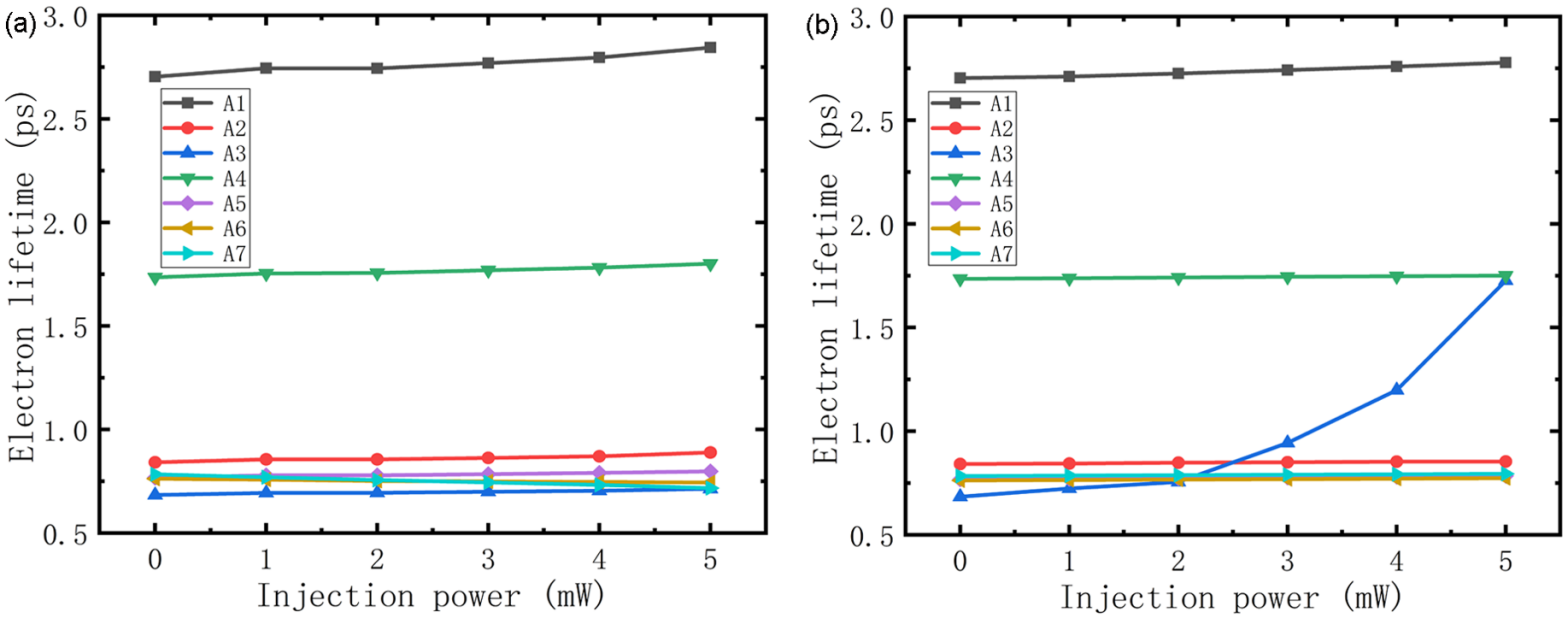
Electron lifetime of each subband using optical injection at wavelengths of 1550 nm (a) and 820 nm (b).

With the variation in electron numbers in each subband, naturally it is necessary to consider the change in electron lifetime in the corresponding subband. As Figure 4a shows, since electrons in subband A1 have the lowest energy compared with those in A2–A7, they have the longest lifetime. The second longest electron lifetime was in A4 as a result of an external electrical field that caused a population inversion. Moreover, in Figure 4a, which depicts the electron lifetime of each subband using injection of light of wavelength 1550 nm, the change in electron lifetime is not very obvious because the number of optically induced electron–hole pairs is much lower (by about an order of magnitude) than for electrons in the cavity. The electron lifetime in A1–A6 increases with injection power because as numbers of electrons increase in those subbands, the number of unoccupied quantum states is reduced, which causes the transition of electrons between those subbands to become less frequent. There are also some differences in Figure 4b. Due to the backfilling effect, which is enhanced by increases in electron temperature, the lifetime of electrons in A3 increases greatly with optical injection power at 820 nm. This behavior results from injected light at that wavelength exciting electrons to high-*k* states, which increases the average temperature of the electrons. And because the backfilling effect only occurs in the lower laser energy level (A3) [20], the variation in electron lifetime with injection power in the other subbands is similar to that in Figure 4a.

### Gain

**Figure 5:**
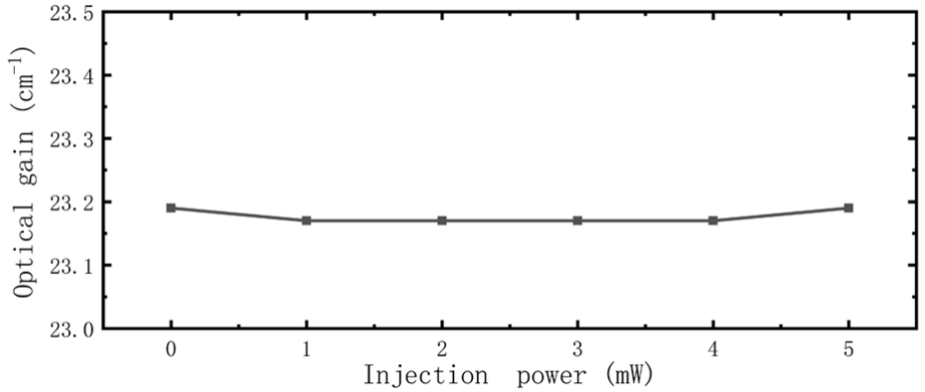
Optical gain as a function of optical injection power.

Optical gain in the cavity during optical injection was also studied. As shown in Figure 5, although the wavelength of injected light is different, the variation of the optical gain is the same and there is less variation. This can be understood from Figure 3 in which the difference in electron number between A4 (upper laser subband) and A3 (lower laser subband) states is almost constant with changes in optical injection power. This difference is indirect proportional to optical gain, indicating that the modulation is not caused by changing the gain.

### Current

**Figure 6:**
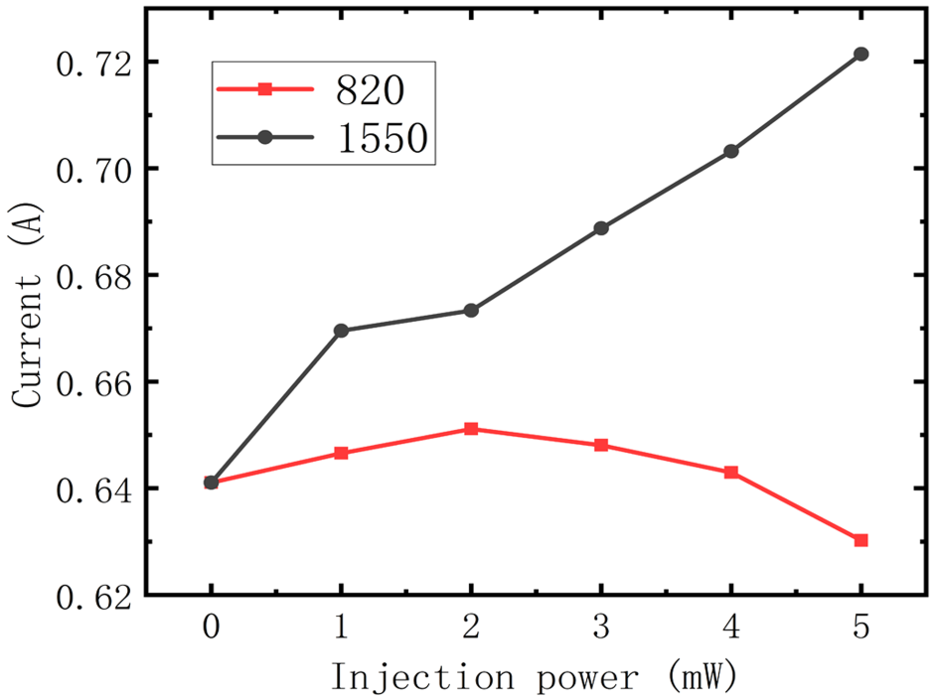
Current vs optical injection power at wavelengths of 1550 nm (black circles) and 820 nm (red squares).

Figure 6 shows when the injection power increases, the cavity current using injected light at a wavelength of 1550 nm increases as well. Because such injected light only increases the number of electrons in the cavity, it enhances the current upon an increase in injection power. While the cavity current using injected light at a wavelength of 820 nm increases at first, when the injection power reaches 2 mW, it reaches its maximum value of 0.65 A and then decreases with further increase in injection power. This behavior occurs because, in addition to increasing electrons in the cavity, injected light at 820 nm also increases the temperature of the electrons, which enhances the backfilling effect and blocks the transition of electrons. Before the injection power reaches 2 mW, the increasing number of electrons plays a dominant role in the process, and the current therefore increases with injection power. Above 2 mW, the effect of backfilling surpasses this effect, so the current is reduced with increasing injection power. In addition, there is an inflection in the curve for injected light at 1550 nm when the injection power reaches 1 mW, where it appears to deviate from a line through the other five points. This results from the arithmetic we used to solve the rate equations. There are numerous solutions to the rate equations, so we solved them iteratively, and for different initial conditions (electron number in the cavity), the solution may not have always been linear.

### Modulation depth

**Figure 7:**
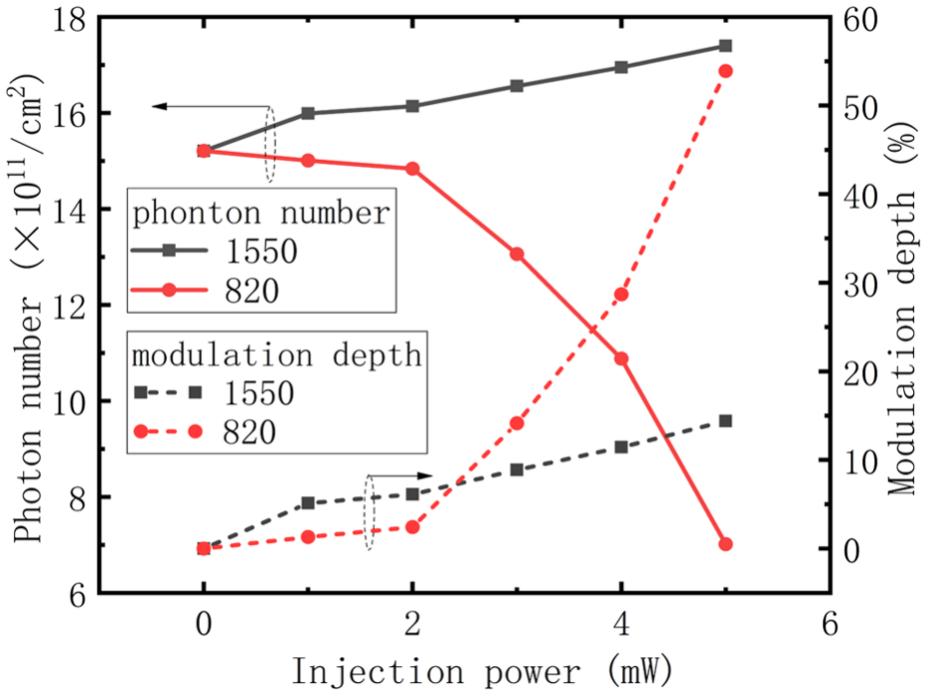
Modulation depth and photon number vs optical injection power at two wavelengths.

Finally, modulation depth using different injection conditions was studied. Figure 5 shows that gain has almost nothing to do with modulation, while for the rate equation, it is not so difficult to demonstrate that the transition speed of electrons between subbands is determined by electron numbers and lifetime in each, and that transition speed is key to the number of photons in the cavity. So we conclude that modulation is induced by the number of electrons and their lifetime. As shown in Figure 7 (black line with square symbols), when the wavelength of the injected light is 1550 nm, the number of photons and modulation depth increase with injection power so that when it reaches 5 mW, the modulation has a value of 15%, which is half the experimental result [19]. The increase in modulation depth with injection power is reminiscent of that for current shown in Figure 6. This modulation characteristic is not difficult to explain, the injected light increases the numbers of electrons (Figure 3a), although it also causes the increase in electron lifetime shown in Figure 4, which may slow the transition. However, from Figure 6 we know that the numbers of electrons play the major role in enhancing current, so with an increase in injection power, the number of photons in the cavity increases, and modulation is achieved. In terms of the injected light at a wavelength of 820 nm shown in Figure 7, the number of photons in the cavity is reduced with an increase in injection power. Although the electron number in A3 and A4 increases as shown in Figure 3b, which enhances the transition process, it can be seen from Figure 4b that electron lifetime in A3 greatly increases, resulting in electrons remaining in A3 for longer times. This blocks the transition from A4 to A3, thus reducing the number of photons in the cavity and achieving modulation.

## Conclusion

In this paper, to explore the phenomenon of all-optical modulation of QCLs, we studied the characteristics of a mid-infrared QCL using near-infrared optical injection of several mW at wavelengths of 820 nm and 1550 nm. A modified classical 1½-period model was established to characterize the process of optical injection, and optically excited electrons and optically induced temperature enhancement are considered in our model. The following parameters were calculated: electron population and lifetime in each subband, optical gain, current and photons in the cavity, which were consistent with the experimental results. We found that for injected light at wavelengths of 820 nm or 1550 nm, electron population and lifetime increases, but does not affect optical gain. Furthermore, injected light at a wavelength of 1550 nm always enhances the current, but at 820 nm only does so when the power of the injected light is greater than 2 mW. Finally, the calculations show that numbers of electrons and electron lifetime in the cavity are of great importance in all-optical modulation of QCLs.
